# Prospective cohort study of HIV incidence and molecular characteristics of HIV among men who have sex with men(MSM) in Yunnan Province, China

**DOI:** 10.1186/1471-2334-13-3

**Published:** 2013-01-04

**Authors:** Junjie Xu, Minghui An, Xiaoxu Han, Manhong Jia, Yanling Ma, Min Zhang, Qinghai Hu, Zhenxing Chu, Jing Zhang, Yongjun Jiang, Wenqing Geng, Lin Lu, Hong Shang

**Affiliations:** 1 From Key Laboratory of AIDS Immunology of Ministry of Health, Department of Laboratory Medicine, No.1 Hospital of China Medical University, Nanjing north street 155#, Heping district, Shenyang 110001, China; 2 From Yunnan Center for Disease Control and Prevention Kunming, Yunnan, China

**Keywords:** Men who have sex with men (MSM), HIV, Incidence, Prospective cohort study, HIV subtypes, Molecular characteristics

## Abstract

**Background:**

Yunnan has the largest number of reported HIV/AIDS cases among all Chinese provinces, the reported prevalence of HIV among Yunnan men who have sex with men (MSM) passed 10%, while HIV incidence epidemic and molecular characteristics of new infected Yunnan MSM were not evaluated before.

**Methods:**

An 18 months prospective followed up with a frequency of 3 month per visit were conducted among HIV seronegative MSM in Kunming cityduring 2009–2011. Interviewer-administrated questionnaires were carried out. Blood specimens were obtained to test for syphilis and HIV, in which HIV were evaluated by standard HIV enzyme immunoassay (EIA) and HIV nucleic acid amplification testing (NAAT). Near full-length regions of the HIV-*1* were evaluated for subtyping, primary drug resistance mutations.

**Results:**

During the follow-up 70.1% of the recruited 378 MSM retained in the cohort. Eleven MSM seroconverted to HIV and fifteen MSM seroconverted to syphilis. The HIV incidence and syphilis incidence was 3.5 (95% CI 1.8-6.2) cases /100 person year(PY) and 5.3 (95% CI 3.0-8.7) cases/100 PY, respectively. Multivariate analysis showed that baseline syphilis infection (aHR, 17.7), occupation (students vs. others [aHR, 5.7], retirees vs. others [aHR, 4.1]), bleeding experience after receptive anal intercourse (aHR,7.6), and minority ethnic(vs. Han) [aHR, 5.7] were independent risk factors for HIV seroconversion(each P<0.05). Among the 7/11 successfully amplified near full-length sequences, 71.4% (5/7) were CRF01_AE, and 28.6% (2/7) were CRF07_BC. Two HIV transmission pairs were detected among seroconverted minority ethnic MSM.

**Conclusions:**

HIV incidence was moderately high among Yunnan MSM. Yunnan province need to strengthen both HIV and syphilis screening among MSM population. Some subpopulations of MSM, such as students, retirees and minority ethnic groups require more HIV epidemic surveillance and strengthened behavior interventions. HIV subtypes and primary drug resistance should be continually monitored to track cross-group transmission of HIV strains.

## Background

In recent years, sexual transmission has become the major route of HIV transmission in China [1,2]. The HIV epidemic among MSM population is of particular concern: homosexually transmitted infection cases accounted for 0.3% of the total reported HIV/AIDS cases in 2006, and rapidly increased to 13.0% in 2011 [1]. In 2008, one large scale cross-sectional study of MSM in 61 cities of China found that the average HIV prevalence had reached 4.9% [2]. Some metropolises such as Beijing [3], Shenyang [4], Harbin [5], Chongqing [6], and Zhengzhou [1]have all seen a rapidly increasing HIV prevalence among their MSM populations.

Yunnan has the largest number of reported HIV/AIDS cases among all Chinese provinces. It accounted for 22% (93,567/429,000) [1,7] of the total reported HIV/AIDS cases (PLWHs) in China. The first outbreak of HIV epidemic was initially detected among injection drug users (IDUs) in Yunnan province, which borders the drug-trafficking routes known as the "Golden Triangle" [8,9]. IDU was the dominant route of HIV transmission route in Yunnan Province during 1989–2006 [10]. However, after 2006, the main HIV transmission route has shifted from IDU to sexual transmission, and the HIV epidemic began spreading from IDUs to other groups [10]. During 2007–2008, the prevalence of HIV among Kunming MSM reached 10.82% [11].

HIV incidence can well reflect the trend of HIV epidemic, and it can also show the effect of HIV intervention efforts. Previously conducted prospective cohort studies in China have revealed the incidence of HIV infection among MSM in Beijing, Nanjing, and Shenyang [12-14]. There is no published report for HIV incidence among Yunnan MSM. Since above prospective surveys were mainly conducted during 2006–2007, they cannot reflect current HIV incidence epidemic among China MSM. Additionally, all of the above three studies only used the testing algorithm of HIV antibody enzyme immunoassay (EIA) combined with western blot(WB) to screen for HIV antibody seroconversion among MSM cohort. Those samples with negative HIV antibody testing results were not tested with pooled nucleic acid testing (NAAT) [12-14], although it has been proven that NAAT can help detect an additional 55% (29/53) of acute HIV infected MSM compared with traditional 3^rd^ generation EIA and WB testing strategy [15].

Another important characteristic of Yunnan Province is its ethnic diversity. Other than the Han ethnic group, 25 minor ethnic groups also reside in the province, accounting for about one third of its total population. During 1989–1995, the reported HIV/AIDS cases in Yunnan province was mainly concentrated in minorities ethnic groups [10]. Since 1996, the Han population has begun to climb in Yunnan and accounted for about 60% of HIV infections in the province until 2006. However, whether minority ethnic groups may have higher HIV incidences rates in Yunnan MSM population was not studied before.

The molecular characteristics of the HIV epidemic can explain the sources of HIV-1 incoming variants and transmission routes. In China HIV/AIDS cases with different HIV transmission routes had quite diverse HIV subtypes. For example, more than 90% of the Yunnan IDUs had CRF_BC subtypes [10], and CRF_AE was the predominant subtype among PLWHs for both homosexual and heterosexual transmission routes [11,16]. Previous molecular analysis of HIV have found the evident of heterosexual transmission of HIV-1 epidemic from Yunnan IDU epidemic [10,16]. However, subtypes, primary drug resistance and other molecular characters of HIV have not been characterized in new HIV infections among MSM in Yunnan.

To objectively evaluate the current HIV incidence among Yunnan MSM, and evaluate the correlation between HIV infection and factors including nationalities, syphilis, illicit drug use behaviors etc., and the HIV subtypes and primary drug resistance of seroconverted MSM, a prospective cohort survey was conducted among MSM in Kunming, the capital of Yunnan Province.

## Methods

### Study design and participants enrollment

Between June 2009 and March 2011, Kunming MSM were recruited to attend an 18 months prospective follow-up with a frequency of three months per visit through a local MSM non-government organization (NGO). The NGO staff approached MSM at various venues and encouraged them to participate in a prospective cohort study screening survey to determine their eligibility for the study.

Each participant was notified before the screening process that they would be followed up and repeatedly tested for HIV and syphilis if they were eligible for the study. The eligible criteria for the cohort entry criteria were: 1) serological negativity for both HIV antibodies and NAAT, 2) at least 18 years of age, 3) having reported to have at least one male sexual partner with whom he had had receptive and/or insentive anal sex in the past 12 months, 4) be physically able and willing to provide written informed consent to participate in the prospective follow-up survey. MSM individuals were interviewed and their eligibility was confirmed through screening questionnaires and HIV testing after they provide written informed consent to the study. Eligible MSM participants were then followed up with a frequency of three months per visit. Interviewed questionnaires were conducted, and blood samples were obtained and tested for HIV and syphilis at each follow-up visit. All MSM participants were provided with general information about HIV/Syphilis transmission and prevention, and were also informed with safe sex practice during pre- and post-test counseling provided in this study. Each participant was compensated with 100 RenMinBi (RMB) [about 16 United States Dollar (USD)] for each round of attending this follow-up. The study protocol and informed consent forms were approved by the Institutional Review Board of the First Affiliated Hospital of China Medical University. Questionnaires surveying on demographics, sexual behavior, illicit drug use, etc. were administered by trained physicians from the Yunnan Provincial Center for Disease Control and Prevention (CDC).

### Laboratory testing

At the baseline stage and during the followup, plasma samples were collected and tested for HIV and syphilis. Plasma specimens were first tested by 3^rd^ EIA assay, Vironostika HIV-1/2 Microelisa System, (BioMe'rieux, Holland). Positive samples were retested using the same EIA assay. HIV positivity was then confirmed by HIV-1/2 Western blot assay (HIV Blot 2.2 WB; Genelabs Diagnostics, Singapore). HIV antibody negative samples were retested with pooled NAAT. A 24-sample mini-pool strategy was adopted to screen for HIV RNA. All MSM individuals with positive NAAT results were followed up to exclude NAAT false-positives [15]. Syphilis was tested using rapid plasma reagin (RPR, Diagnosis; Shanghai Kehua, China). Plasma samples with positive RPR results were retested by Treponema pallidum particle assay (TPPA, Serodia, Japan). Participants whose plasma samples showed positivity in both TPPA and RPR were determined to be currently infected with syphilis.

### HIV Molecular analysis

Ribonucleic acid(RNA) was extracted from plasma and used to synthesize cDNA as previously described [17]. The 3' or 5' halves of viral cDNA were separately amplified by single genome amplification and sequencing [18]. Individual sequence fragments for each amplicon were assembled and edited using the sequencher program (version 4.9). All nucleotide sequences were aligned using the CLUSTAL X program followed by manual editing with various HIV-1 reference subtypes and circulating forms (CRFs) downloaded from the Los Alamos HIV Sequence Database using Bioedit software (available at URL: http://www.mbio.ncsu.edu/bioedit/bioedit.html Accessed 30 January 2013). The phylogenetic analyses were performed using the neighbor-joining method in MEGA version 4.0 for subtyping analysis. Mutation profile and predicted genotypic resistance were analyzed by comparison to the Stanford HIV Drug Resistance Database (hivdb.stanford.edu).

### Data analysis

Questionnaires responses were double entered and then checked for accuracy using Epi Data software (The Epi Data Association Odense, Denmark, version 3.02). Data were then analyzed using SAS 9.1 (SAS Institute Inc., Cary, NC). HIV seroconversion was estimated to have occurred at the midpoint between the time of baseline HIV-1 test and the time of the follow-up HIV-1 test with a seropositive result. HIV incidence density was calculated based on a Poisson distribution, with person-year (PY) over the entire follow-up period as the denominator. Multivariate Cox proportional hazard regression model was used to determine the adjusted hazard ratio(aHR) aHRfor HIV seroconversion related factors. Marginally significant variables with P< 0.25 in univariate analysis were included in multivariate analysis. Only the variables with P< 0.05 were kept in the last stepwise multivariate model.

## Results

### Demographic characteristics of the MSM cohort

A total of 423 MSM were approached to attend the eligibility screening, in which 43 were found to be HIV positive and 2 declined to attend the follow-up, thus were excluded from the prospective cohort study. A total of 378 eligible MSM were recruited to participate in the prospective cohort study. The median age was 28 years, with 50.8% of the cohort younger than 28 years. 69.3% were single and 14.0% were married with female. 82.3% were Han ethnic and 17.7% were minority ethnic. For education level, 45.5% had received lower than junior high school education, and 24.3% had received college-level or higher education. The main routes to seek homosexual partners were through the internet (43.4%), the bar/night club/tearoom (15.9%), the park/public restroom/square (13.5%), and through Park/public restroom (7.9%). In terms of occupations, 16.4% were male students, 12.4% were retirees, and 71.2% were none-students or retirees; Comparison analysis of characteristics between MSM participants who lost to follow-up and those retained in the cohort showed that, the distribution of current ages, permanent residence locations, sexual orientations, and main location to seek homosexual partners were statistically significant different between (each <0.05) (Table 1).

**Table 1:** Baseline demographic characteristics of Kunming MSM participants

**Characteristics**	**No. of lost to follow-up MSM (proportion,%)**	**No. of MSM retained in cohort (proportion,%)**	**Total No. Of MSM(proportion,%)**
Age (yrs.)			
≥30	75(66.4)	126(47.5)**	201(53.2)
18-29	38(33.6)	139(52.5)	186(46.8)
Marriage status			
Single	86(76.1)	177(66.8%)	263(69.6)
Married	10(8.8)	43(16.2%)	53(14.0)
Cohabitating	14(12.40%)	28(10.6%)	42(11.1)
Divorced/widowers	3(2.7%)	17(6.4%)	20(5.3)
Permanent residence			
Kunming City	23(20.4%)	113(42.6%)***	136(36.0)
Other cities in Yunnan province	49(43.4%)	88(33.2%)	137(36.2)
Other provinces	41(36.3%)	64(24.2%)	105(27.8)
Nationality			
Han	90(79.6)	220(83.0%)	311(82.3)
Non-Han	23(20.4)	45(13.0)	67(17.7)
Level of education			
College and above	42(37.2%)	130(49.1%)	172(45.5)
Senior high school	34(30.1%)	80(30.2%)	114(30.2)
Junior high school and below	37(32.7)	55(20.7)	92(24.3)
Sexual orientations			
Homosexual orientation	47(41.6%)	164(61.9%)**	211(55.8)
Bisexual orientation	64(57.9)	101(38.1)	165(43.7)
Heterosexual orientation	2(0.5)	0(0.0)	2(0.5)
Main location to seek homosexual partners			
Internet	29(25.7%)	31(11.7%)*	164(43.4)
Bar/night club/tearoom	6(5.3%)	24(9.1%)	60(15.9)
Park/public restroom/square	14(12.4%)	37(14.0%)	51(13.5)
Bathing room/sauna/massage room	47(41.6%)	117(44.2%)	30(7.9)
Other places	17(15.0%)	56(21.1%)	73(19.3)
Occupations			
Others	82(72.6%)	187(70.6%)	269(71.2)
Students	18(15.9%)	44(16.6%)	62(16.4)
Retired	13(11.5%)	34(12.8%)	47(12.4)

*, P<0.05; **,P<0.01; ***,P<0.001.

### Migrant status of MSM participants

The participants’ registered residency showed that 36.0% (136/378) of participants were registered to reside in Kunming city, 36.2% (137/378) were from other cities in Yunnan Province; and 27.8% (105/378) were from outside of Yunnan province. Total 21.9% (53/242) migrant participants were minority ethnic groups from outside of Kunming city. Within those from outside of Kunming city, 60.3% (146/242) lived for more than two years in Kunming city, and 39.7% lived for less than two years.

### Sexual behaviors, illicit drug use and other HIV related factors

The average age of first sexual intercourse was 20.4 ± 4.5 years. 216 (57.1%) participants had insertive anal intercourse with a male sexual partners and 162 (42.9%) had vaginal intercourse with a female partner. The median age to have sex with a male partner for the first time was 23 years. Eleven (5.1%) participants were older than 40 years of age when they had sex with a male partner for the first time.

In the most recent six months prior to the time of the questionnaire survey, 95.5% (361/378) of the participants had anal sex with other male sexual partners, among whom 32.4% had one male partner, 40.4% had 2–3 male sexual partners, 11.6% had 4–5 male sexual partners, and 15.5% had more than 5 male partners. In the course of anal sex with males 48.9% (185/361) constantly used condoms, 36.0% (136/361) occasionally used condoms, and 10.6 (40/361) never used condoms. 66.1% (250/378) of participants had consistent male sexual partners, among whom 51.6% failed to use condoms during anal intercourse. 57.4% (217/378) had casual partners, among whom 29.6% failed to use condoms during anal intercourse. More than one quarter 69 (18.3%) experienced anal bleeding after receptive anal intercourse, and 60 (15.8%) experienced condom breakage or slippage. 47 (12.4%) purchased sex from male partners and 14 (4.2%) solicited sex. About one quarter of the participants 91 (24.1%) had engaged in heterosexual behavior, among whom 55 (60.4%) failed to use condoms during virginal intercourse with female sex partners.

Very few 12 ( 3.2%) reported to have ever used any of the listed illicit drugs (opium, heroin, methamphetamine, morphine, cannabis, cocaine, dolantin, ketamine, triazolam, or oramphetamine), and only 8.3% (1/12) once injected drugs, but none reported to have shared needles with other injection drug users. Forty-four (11.6%) of the participants were circumcised.

### Baseline prevalence of syphilis among MSM participants

Among the recruited 378 HIV negative MSM cohort, all provided blood samples and the tested prevalence of syphilis was 5.8% (22/378).

### HIV/syphilis incidence and HIV seroconversion related major factors

During the follow-up, 70.1% (265/378) of the MSM were retained in the cohort, with the total accumulated follow-up person time being 312.1 person-year (PY). Totally 11(2.9%) MSM became HIV seroconverted, in which seven MSM were found with HIV antibody seroconversion and four MSM were detected to have acute HIV infection by NAAT testing during the follow-up. The calculated HIV incidence density was 3.5 (95% CI 1.8-6.2) cases /100 PY. Fifteen participants seroconverted to syphilis during a total of 279.1 PYs follow-up, the calculated syphilis incidence density was 5.3 (95% CI 3.0-8.7) cases /100 PY.

Multivariate Cox hazard regression analysis showed that baseline syphilis infection (aHR], 17.7, 95% CI, 3.6-86.4, P<0.001), occupation (male students vs. others [aHR, 5.7, 95% CI, 1.3-24.3, P=0.019], retirees vs. others [aHR, 4.1, 95% CI, 1.0-18.6, P=0.05]), bleeding experience after receptive anal intercourse in past 6 months (aHR, 7.6, 95% CI, 2.2-26.6, P=0.001), and ethnicity (ethnic minorities vs. Han) [aHR, 5.7, 95% CI, 1.5-21.5, P=0.01] were independent risk factors for HIV seroconversion among the recruited MSM cohort (Table 2).

**Table 2:** Univariate and multivariate analyses of participants characteristics associated with incident HIV infection among Kunming MSM (N=378)

**Factors**	**No. of HIV seroconversion**	**Cumulative PY**	**HIV incidence(/100PY)**	**Hazard ratios (95% CI)**	
				**Univariate**	**Multivariate**
Age(years)					
≥30	4	143.2	2.8		
18-29	7	169.0	4.1	2.0(0.6-6.9)	-
Ethnicity					
Han	6	262.8	2.3		
Non-Han	5	49.3	10.1	4.2(1.3-13.8)	5.7(1.5-21.5)
Occupations					
Other occupations	4	218.3	1.8		
Male students	4	51.7	7.7	4.1(1.1-16.3)	5.7(1.3-24.3)
Male retirees	3	42.1	7.1	4.0(1.0-17.8)	4.1(1.0-18.6)
Preferred major sexual roles with male sexual partners					
Single insertive anal sexual role	1	125.7	0.8		
Receptive or both insertive and receptive anal sexual role	10	186.4	5.4	6.6(0.8-51.6)	-
Main location to seek homosexual partners					
Bar/night club/tearoom	2	35.1	5.7		
Bathing room/sauna/massage room	3	25.3	11.9	2.2(0.4-13.0)	-
Park/public restroom/square	0	44.4	0.0	NA	-
Internet	5	139.6	3.6	0.6(0.1-3.1)	-
Other ways	1	67.7	1.5	0.4(0.0-2.8)	-
The number of occasional male sexual partners in recent 6 months					
0-1	5	168.3	3.0		
>1	6	143.8	4.2	1.5(0.4-4.8)	-
Bleeding experience after receptive anal intercourse					
No	5	262.6	1.9		
Yes	6	49.6	12.1	6.1(1.9-20.1)	7.6(2.2-26.6)
Condom using conditions for the last receptive anal intercourse					
No	6	239.5	2.5		
Yes	5	72.6	7.7	2.8(0.9-9.3)	-
Buying sex from males in recent 6 months					
No	10	296.2	3.4		
Yes	1	15.9	6.3	1.9(0.2-14.5)	-
Selling sex from males in recent 6 months					
No	1	301.8	0.3		
Yes	0	10.4	0.0	NA	-
Illegal drug using behaviors					
No	11	304.0	3.6		
Yes	0	8.2	0.0	NA	-
Male circumcision					
No	9	213.5	4.2		
Yes	2	98.6	2.0	0.8(0.1-6.0)	-
Syphilis infection					
No	8	291.6	2.7		
Yes	3	20.5	14.6	5.6(1.5-21.3)	17.7(3.6-86.4)
Failed to use condom with regular male sexual partners during receptive anal sex in the follow-up					
No	2	94.3	2.1		
Yes	9	217.7	4.1	1.9(0.4-8.8)	-
Failed to use condom with regular male sexual partners during receptive anal sex in the follow-up					
No	1	96.9	1.0		
Yes	10	215.2	4.6	4.4(0.6-34.7)	-

NA: None applicable; CI, confidence interval; PY, person year.

### HIV subtyping and primary drug resistance genotyping

**Figure 1:**
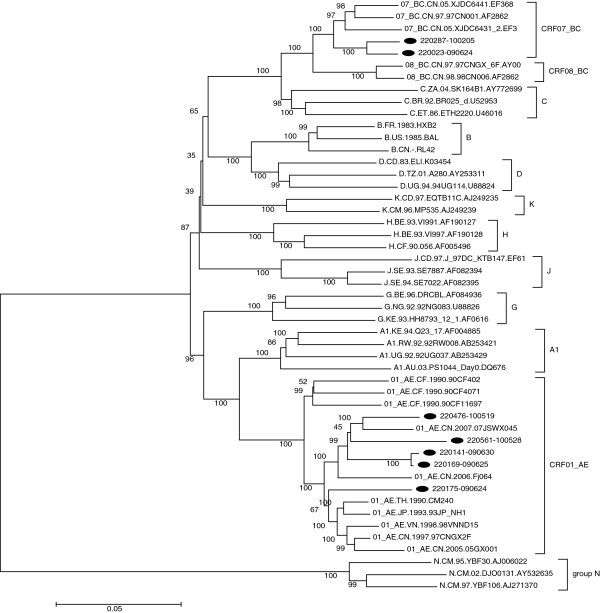
Phylogenetic tree analysis of near full-length nucleotide sequences from newly infected MSM in Yunnan identifies.

**Figure 2:**
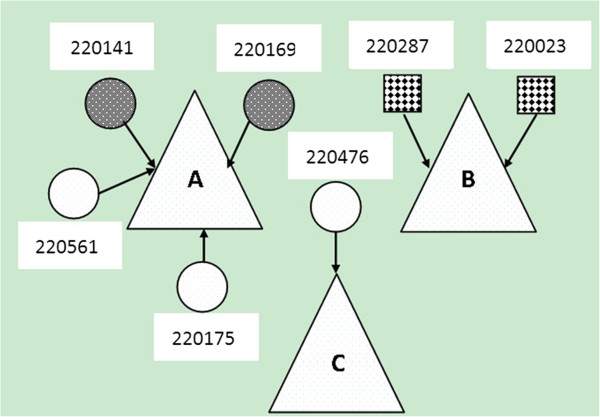
**Network diagram of 7 HIV seroconverted MSM and the detected HIV transmission pairs.** The three triangles in the diagram represent main locations in which MSM seek homosexual partners(triangle **A**: Internet; triangle **B**: Bathing room/sauna/ massage room; triangle **C**: Bar/night club/tearoom). The seven circles represent HIV antibody seroconverted MSM with the HIV-1 subtype of CRF01_AE and the two rectangles represent MSM with the HIV-1 subtype of CRF_07BC. Two HIV transmission pairs ([220287 vs. 220023] and [220141 vs. 220169]) were detected based on the phylogenetic analyses method. Their transmission relationship was confirmed by further conducted epidemiological survey.

A total of 7 HIV-1 nucleotide sequences of near full-length (HXB2: 769-9384nt) were successfully amplified and determined from eleven identified seroconverted Yunnan MSM, using SGA method. Their estimated median time of HIV infection was 69 days, and none once took Highly Active Antiretroviral Therapy (HAART) before attending this survey. The distribution of HIV-1 genotypes determined based on neighbor-joining tree analyses was as follows (Figure 1): CRF01_AE, 71.4% (5 of 7) and CRF07_BC, 28.6% (2 of 7). By phylogenetic comparison of virus sequence, we found two HIV transmission pairs, in which two MSM participants (220287 and 220023) had closely related CRF_BC and another two MSM participants (220141and 220169) had closely related CRF_AE. Their transmission relationship was confirmed by later conducted epidemiological survey, for they both reported once had unprotected anal intercourse before this study, and all of the four participants belonged to Yi ethnic, all of the two transmission pairs went to the same venues to seek sexual partners. While, none of them once injected drugs before (Figure 2). No mutation against protease inhibitors (PIs) was detected. Non-nucleoside reverse transcriptase inhibitor (NNRTI)-associated mutation G190E with high-level resistance to nevirapine (NVP)and efavirenz (EFV) was seen in one subtype CRF01_AE sequence (14.3% ,1/7). V179E, which combined with G190E decreases susceptibility to etravirine (ETR) was also found in the same patient.

## Discussion

This study firstly evaluated the HIV incidence and the molecular characteristics of HIV seoconverted MSM among Yunnan MSM by prospective survey. The HIV incidence among Kunming MSM is moderately high: it is higher than the HIV incidence among Beijing MSM population (2.6/100 PY [14], while lower than the HIV incidence among Shenyang MSM (5.4/100 PY) [12] and Nanjing MSM (5.1/100 PY) [13]. And it is comparable with the HIV incidence among MSM in the Netherlands [19], and the United States [20,21]. Although at present heterosexual acts is still the dominant transmission route among recently reported HIV/AIDS cases, the proportion of MSM involved cases has increased rapidly. Without effective intervention, the HIV prevalence may continue to increase among Kunming MSM, and MSM may possibly become the largest demographic group in newly reported HIV infections cases, similar to what has been observed in European countries and America [22,23].

Illicit drug abuse is very common among the MSM populations in America [20,24,25]. In this study, the prevalence of illicit drug abuse behavior is 3.2%, which is relatively higher than that in Beijing (0.8%) [14] and Shenyang (2.8%) [12], while it is significantly lower than Chengdu MSM [26]. Only 1 out of the 12 illicit drug users in the cohort reported once injected drug, and no one admitted to have shared needles with other IDUs, which partially explained why illicit drug abuse is not significantly correlated with HIV seroconversion among these MSM participants. Similarly, none of the previous prospective cohort studies in China found any correlation between illicit drugs and HIV incidence [12-14]. Only one recent study to Shenyang MSM found a positive correlation between illicit drug use behavior and HIV infection status [27]. Nonetheless, Illicit drug use should be continually monitored among Kunming MSM population, since the possibility of it promoting the HIV epidemic cannot be eliminated.

This study found that syphilis infection, ethnicity (Minorities vs. Han), occupations (students/ retirees vs. other occupations) and bleeding experience after receptive anal intercourse were independently correlated with HIV infection. The baseline prevalence of syphilis is only 5.8%, lower than the syphilis prevalence among MSM in Shenyang (25.4%) [12], Beijing (19.8%) [28] and Nanjing(12.3%) [13]. But HIV seoconversion risk among syphilis-infected Kunming MSM is 17.7 times higher than that among the syphilis negative participants. This and the previous cohort studies involving high risk MSM revealed that syphilis infection was a common risk factor for promoting HIV infection among Chinese MSM. This discovery provides important supportive information to implement a syphilis screening and treatment program for MSM populations at the voluntary counseling and testing (VCT) sites across China. This information can also help decrease HIV acquisition rate and transmission risk among this population.

In this study, four acutely infected MSM individuals with initial HIV antibody-negative were detected through NAAT, accounting for almost forty percent of all the seroconverted MSM participants in this study. If these acutely infected participants had exited the study in the middle of the follow-up, they could have been unidentified based on the traditional HIV testing strategy alone, causing an underestimate of HIV incidence in the recruited cohort. Considering its efficiency and cost-effectiveness [15,29] for AHI screening among MSM, NAAT should be considered to be integrated into the HIV testing strategies for HIV incidence estimations and behavior intervention in future MSM cohort studies where HIV incidences were relatively high.

This study found that among MSM population the subpopulation of students and retired males both had significantly higher risk for HIV seroconversion than other MSM populations. This may partially explain why the people who are 50 years older and students have occupied an increasing proportion of the total reported HIV cases in China [2]. Additionally, it also explains the male-to-female ration among reported national over 65 years people PLWHs increased rapidly in recent years [30]. Similar evidence was also reported among MSM students by recent study, which found that unprotected homosexual behavior accounted more and more HIV infection among reported HIV infection cases in China, male-to-male homosexual route infection accounted for 17.5% of the total reported student HIV infection cases in 2006, but this figure rapidly increased to 62.4% in 2009 [31]. Our study result further shows that Yunnan MSM students and retired MSM are particularly vulnerable population to HIV infection compared with other MSM populations, large scaled HIV screening should be prioritized for these group [32]. Additionally, these subpopulations should receive more attention in future HIV epidemic surveillance and behavior intervention efforts.

This study also revealed a significantly higher HIV incidence among minority ethnic MSM comparing with Han MSM in Yunnan Province. This observation indicated that HIV prevention and intervention efforts should be implemented not only in big cities, but also in more remote districts in Yunnan Province, since the province’s demography has a significantly higher ethnic minority proportion than other provinces in China. Furthermore more than one fifth of these participants in this study were migrants minority MSM from outside of Kunming city, which showed that more health education and behavior intervention effort should be implemented to decrease their HIV acquisition risk among Yunnan minority ethnic MSM.

The subtype analysis in our study found that 71.4% of the HIV-strains were CRF01-AE and 28.6% were CRF07_BC among newly HIV-infected MSM in Yunnan. This is consistent with the results of a recent cross-sectional study on HIV subtypes among Yunnan MSM [32]. For CRF_BC was the traditionally predominant HIV subtypes among Yunnan IDUs [10,33,34] and, and CRF_AE was the traditional major HIV subtypes among Yunnan MSM [32], so the appearance of both subtypes CRF01-AE and CRF07_BC among these seroconverted MSM may reflect the new trend of HIV transmission from IDU to MSM populations. Therefore continuing monitoring will be helpful to track cross-group transmission among MSM populations, for example, from local IDUs to MSM. For HIV primary drug resistance testing, no primary mutations to PI were observed, but one MSM had RT-associated mutation G190E with high-level resistance to NNRTIs and V179E with reduced susceptibility to NRTIs which may be the first report of the primary drug resistance in the early infection stage in the infected MSM population in Yunnan.

There were some limitations in this study. Firstly, the cohort retention rate was moderate (70.1%), which is higher than or similar to the retention rate in other cohort surveys on MSM in Netherlands (65.6%) [35], Shenyang (56%) [12] and Nanjing (72%) [13], while lower than that of a Beijing cohort study (86.2%) [14]. Secondly, some characteristics of those lost to follow-up and those retained in the cohort were statistically significant different, which may weaken the generalizability of the study findings to total Kunming MSM. Thirdly, the size of the MSM cohort is moderate. While larger than that in the Dutch cohort study (190) [35] and the Shenyang cohort (n=218) [12], and similar to Nanjing cohort (n=397) [13], but it is smaller than the Beijing cohort (n=507) [14]. A large sample size is statistically more representative and can more accurately reflect HIV incidence in the overall MSM population at a particular location. Fourthly, because illicit drug abuse is a sensitive topic for participants, so this study cannot exclude the likelihood of under reporting illicit drug using rate for social desirability bias [36]. Lastly, eleven MSM seroconverted to HIV during the follow-up, while in which only 7 HIV-1 nucleotide sequences of near full-length were successfully amplified and determined, the relative small sample size may decrease the statistical power in evaluating the distribution of HIV-1 genotypes among newly HIV infected MSM.

## Conclusions

HIV incidence was moderately high among Yunnan MSM, where venous injection drug use was the traditionally dominant HIV transmission route. Yunnan province needs to strengthen both of HIV and syphilis screening program among this population. Some subpopulations of MSM, such as students, retirees and minority ethnic groups require more HIV epidemic surveillance and strengthened behavior interventions. HIV subtypes and primary drug resistance should be continually monitored to track cross-group transmission of HIV strains.

## Competing interest

This study was supported by the Mega-projects of national science research for the 12th Five-Year Plan (2012ZX10001-006); China-Gates Foundation Cooperation Programme; and National Nature Science Foundation of China (81001291). The funding organization had no role in the development of study design or in the collection, analysis, and interpretation of data. The authors declare that they have no competing interests.

## Authors’ contributions

Conceived and designed the experiments: JJX LL HS participated in the design of the study. MHA XXH MHJ YLM MZ performed the study and experiments. JJX MHA analyzed the data; QHH ZXC JZ YJJ WQG contributed reagents/materials/analysis tools; JJX HS LL wrote and revised the manuscript. All authors read and approved the final manuscript.

## Pre-publication history

The pre-publication history for this paper can be accessed here:

http://www.biomedcentral.com/1471-2334/13/3/prepub
